# Cardiopulmonary fitness, insulin sensitivity and myocardial fat: a CMR/MRS study in obese non-diabetic women

**DOI:** 10.1186/1532-429X-13-S1-O80

**Published:** 2011-02-02

**Authors:** Wolfgang J Utz, Sven Haufe, Martin Pofahl, Julius Traber, Friedrich C Luft, Jens Jordan, Jeanette Schulz-Menger

**Affiliations:** 1 Working Group Cardiac MR, Medical faculty of the Charité and HELIOS Klinikum Berlin Buch, Berlin, Germany; 2 Franz Volhard Clinical Research Center at the Experimental and Clinical Research Center, Charité and Max Delbrück Center for Molecular Medicine, Berlin, Germany, Berlin, Germany; 3 Institute of Clinical Pharmacology, Hannover Medical School, Hannover, Germany, Hannover, Germany

## Background

Obesity predisposes to heart failure, particularly in sedentary subjects. Among others, alterations in myocardial substrate metabolism, myocardial triglyceride (MTG) accumulation and lipotoxicity may be involved. MTG is excessive in overt diabetes and related to diastolic dysfunction. In men, exercise training improves MTG, cardiac performance, physical fitness and insulin sensitivity. Given the gender difference in metabolic response to obesity these results cannot be simply extrapolated to women. Thus, it was the objective to explore the relation between cardiorespiratory fitness, cardiac function, and MTG in obese women.

## Methods

**Figure 1:**
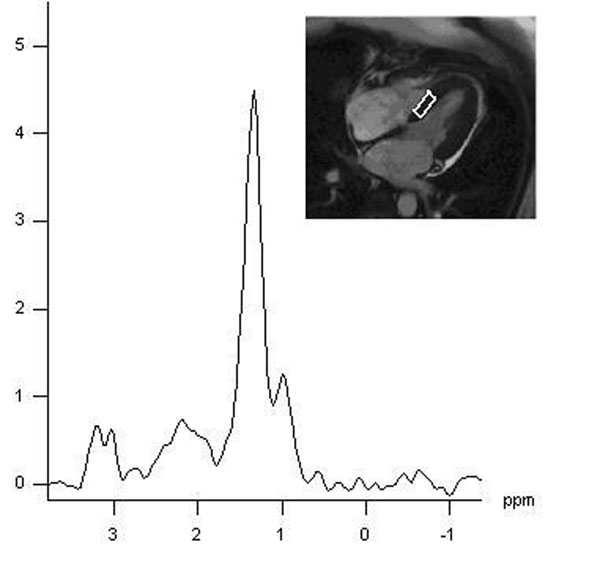


Sixty-five overweight/obese, but otherwise healthy women were included in the study (BMI 33±4 kg/m^2^; 45±10 yrs). Insulin sensitivity given as composite insulin sensitivity index (c-ISI) was determined by oral glucose tolerance testing and maximum whole-body oxygen uptake as a measure of cardiopulmonary fitness by spiroergometry. Cardiac function was measured by temporal high resolution CMR cine imaging (64 phases, TR = 16.3 ms) on a 1.5T clinical MR scanner and diastolic function was derived from volume-time curves as ratio of peak filling rates in early and atrial phase (PFR_E_/PFR_A_). MTG was determined by dually gated ^1^H single voxel spectroscopy (SVS) from a 6-8x20x25 mm^3^ voxel in the interventricular septum (Figure 1) and triglyceride levels in the skeletal muscle (Tibialis anterior muscle) by ungated ^1^H SVS.

## Results

**Figure 2:**
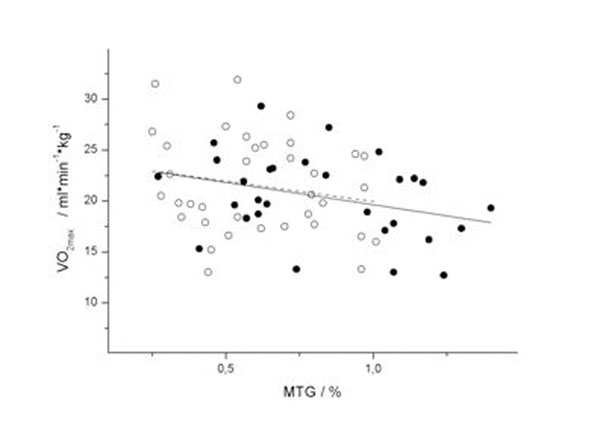


MTG content was negatively correlated with cardiopulmonary fitness (r=-0.28, p=0.02) in bivariate correlation analysis (Figure 2). In women with prediabetic (c-ISI < 4.5, n=29) vs. normal glucose tolerance (c-ISI > 4.5, n=36), MTG content was higher (0.83±0.30% vs. 0.61±0.23%, p=0.002) and cardiac diastolic function was impaired (1.32±0.44 vs. 1.55±0.46, p=0.047). Age, 24h blood pressure and cardiopulmonary fitness (20±4 ml*min^-1^*kg^-1^ vs. 22±5 ml*min^-1^*kg^-1^, p=0.32) were equal in both groups.

In contrast, triglyceride levels in the skeletal muscle did not correlate to cardiopulmonary fitness in the study group (p>0.05), but correlated negatively with insulin sensitivity (r=-0.32, p=0.02).

## Conclusions

In overweight and obese women, reduced cardiopulmonary fitness is associated with increased myocardial triglyceride content. Prediabetic glucose tolerance in obese females is associated with higher MTG and reduced diastolic function. Exercise-based life-style interventions could have a beneficial effect on cardiac function through myocardial triglyceride mobilization.

